# Improving the Use of the Mental Health Risk Assessment (MHRA)

**DOI:** 10.1192/bjo.2022.500

**Published:** 2022-06-20

**Authors:** Libby Sampey, Dennis Walzl, Colette Wee, Robert Clafferty

**Affiliations:** 1 Royal Edinburgh Hospital, Edinburgh, United Kingdom; 2 University of Edinburgh, Edinburgh, United Kingdom

## Abstract

**Aims:**

The MHRA is a comprehensive form on our electronic patient records system. It includes 11 sections assessing different risk categories, with tick boxes to evidence input from various members of the MDT. Anecdotal experience suggested that these forms were sometimes incomplete and often lacked input from MDT members other than nursing staff. We aimed to increase the completion rate and multidisciplinary team (MDT) involvement, particularly doctor involvement, in the electronic MHRA documentation on an acute inpatient psychiatric assessment ward at the Royal Edinburgh Hospital.

**Methods:**

- Baseline survey (November cohort of 12 patients): data collection on number of sections completed (total number = 11) and whether the ‘psychiatrist’ box was ticked, indicating medical input.
- Intervention: doctors on the ward reviewed all inpatient MHRAs, added additional assessments if appropriate, and ticked ‘psychiatrist’ involvement in the MHRA.
- Repeat survey (February cohort of 11 patients): data collection as before and review of findings.

**Results:**

In our baseline survey (November 2021), 75% (9/12) of patients had all sections of the MHRA completed. 33% (4/12) had the ‘psychiatrist’ box ticked. In our repeat survey (February 2022), 91% (10/11) of patients had all sections of the MHRA completed. 100% (11/11) had the ‘psychiatrist’ box ticked.

**Conclusion:**

Accurate assessment and management of risk is an important factor in the safety of patients and staff on acute psychiatric wards. Our baseline data showed that risk assessments had limited medical input and at times had sections which were not filled in at all. Review of the MHRA by medical staff improved this, and in some cases found and added relevant information which had been missed. As a person dependent intervention, this may not be a sustainable change. As a first step to introduce a sustainable system change, a visual prompt has been introduced, in the form of a blue triangle icon in the duty room whiteboard to highlight whether each patient has a complete and up to date MHRA. Further interventions could include integrating a review of the MHRA in weekly ward rounds. This audit also raised the issue of some relevant information having been missed from risk assessments and showed that further audit of the quality of risk assessments is indicated.

